# Quantifying Urban Spatial Variations of Anthropogenic VOC Concentrations and Source Contributions with a Mobile Sampling Platform

**DOI:** 10.3390/ijerph16091632

**Published:** 2019-05-10

**Authors:** Peishi Gu, Timothy R. Dallmann, Hugh Z. Li, Yi Tan, Albert A. Presto

**Affiliations:** Center for Atmospheric Particle Studies, Department of Mechanical Engineering, Carnegie Mellon University, Pittsburgh, PA 15213, USA; peishigu@gmail.com (P.G.); trdallmann@gmail.com (T.R.D.); hughli.netl@gmail.com (H.Z.L.); solartan@gmail.com (Y.T.)

**Keywords:** air toxics, source apportionment, BTEX, mobile sampling

## Abstract

Volatile organic compounds (VOCs) are important atmospheric constituents because they contribute to formation of ozone and secondary aerosols, and because some VOCs are toxic air pollutants. We measured concentrations of a suite of anthropogenic VOCs during summer and winter at 70 locations representing different microenvironments around Pittsburgh, PA. The sampling sites were classified both by land use (e.g., high versus low traffic) and grouped based on geographic similarity and proximity. There was roughly a factor of two variation in both total VOC and single-ring aromatic VOC concentrations across the site groups. Concentrations were roughly 25% higher in winter than summer. Source apportionment with positive matrix factorization reveals that the major VOC sources are gasoline vehicles, solvent evaporation, diesel vehicles, and two factors attributed to industrial emissions. While we expected to observe significant spatial variability in the source impacts across the sampling domain, we instead found that source impacts were relatively homogeneous.

## 1. Introduction

Volatile organic compounds (VOCs) are emitted to the atmosphere by both natural and anthropogenic sources [1]. They are an important part of the atmospheric oxidation processes that generate ozone and secondary organic aerosols (SOA) [2,3]. Many VOCs are also known to have negative health effects. A majority of the 187 compounds classified as hazardous air pollutants (HAPs) by the U.S. EPA are VOCs [4]. 

Important anthropogenic sources of VOCs include vehicular emissions, oil and gas operations, industrial processes, evaporation of volatile organic solutions, and consumer products [5]. Two important classes of anthropogenic VOCs are aliphatic hydrocarbons (such as alkanes, alkenes, etc.) and aromatics (such as benzene, toluene, ethylbenzene and xylenes, also known as BTEX) [6,7,8,9]. The BTEX species are all HAPs. For example, numerous cohort studies and other epidemiology studies found correlation of benzene exposure to adverse health effects [10,11,12,13,14,15,16,17].

In the U.S., long-term monitoring of VOCs is sparse compared to criteria pollutants such as fine particulate matter (PM_2.5_) and O_3_. The U.S. National Air Toxics Trend Stations (NATTS; https://www3.epa.gov/ttnamti1/natts.html) monitor long-term trends in toxic VOCs in 27 primarily urban locations [1]. Such datasets are crucial to inform the inter-city differences and long-term trends of VOC concentrations, but cannot quantify intra-city variabilities due to the limited number of monitoring sites within each city. 

Most air pollutants exhibit intra-city variations, driven primarily by emissions from local sources [18,19,20,21,22]. Elevated concentrations of particulate black carbon (BC), carbon monoxide, and nitrogen oxides near roadways have been extensively characterized [18,20]. While PM_2.5_ mass is dominated by secondary components, concentrations of primary PM_2.5_ and specific PM components can also be elevated near emissions sources such as industrial facilities and restaurants [19,20,21,22]. 

VOCs, especially those emitted from anthropogenic sources, also exhibit intra-city spatial variations. Since oxidative lifetimes of many anthropogenic VOCs (e.g., BTEX) are significantly longer than timescales of dilution and dispersion, we expect intra-city VOC spatial patterns to be driven by the location and intensity of emissions sources. Several studies investigated the intra-city spatial variability of benzene using distributed monitoring campaigns [10,23,24,25]. Often these sampling campaigns are limited in spatial and/or seasonal coverage [24]. Spatial modeling tools such as the EPA National Air Toxics Assessment (NATA) [26,27,28], chemical transport models including the Community Multiscale Air Quality (CMAQ) model [29], and land-use regression [13,25,30,31,32] have all been used to investigate intra-city variations in BTEX exposure [25,29]. Overall, spatial patterns of VOCs have received less attention than pollutants such as PM_2.5_ and NO_2_, so less is known about urban patterns of VOC exposure and the sources driving those spatial patterns.

In this study, we utilized a mobile sampling platform with an on-board gas chromatograph with flame ionization detection (GC-FID) to characterize VOC concentrations in Pittsburgh, PA. We performed measurements in more than seventy locations in the urban area to examine the magnitude of spatial variability of VOC and BTEX concentrations. We applied two source apportionment techniques to identify the important sources in the region and the contribution of each source to the total VOC concentration. Previously, Tan et al. [18] published part of the benzene and toluene measurement results from the first phase of this project. This study is an extension and a more in-depth investigation of spatial variability in concentration and source contributions to VOCs.

## 2. Materials and Methods 

### 2.1. Data Collection

We collected all of our data in Pittsburgh and surrounding areas in Allegheny County, Pennsylvania, USA from 2011 to 2014. The field measurement was planned and conducted in two phases, with each phase covering a different list of sites that were selected from distinctive areas in order to capture emissions from various types of sources. In each phase, data was collected in both winter and summer. In each season, each site was visited during morning (6 a.m. to 11 a.m.), evening (3 p.m. to 9 p.m.) and night-time (12 a.m. to 5 a.m.) periods so that the diurnal variation of the pollutant concentrations could be captured. 

Table 1 summarizes the instruments used in each phase of measurements. We used a compact Chromatotec Airmo BTX GC866 (Chromatotec America Inc: Houston, TX, USA) to measure VOC concentrations. The instrument ran semi-continuously on a 15 min cycle, and during each cycle the instrument sampled from the ambient for 11 min with a working flow rate of 50 mL/min. The detection limit of this instrument is 0.2 µg/m^3^ and the uncertainty is 3%. More information about the instruments and the mobile lab are available in previous literature [18,33].

#### 2.1.1. Phase I Measurements

**Figure 1:**
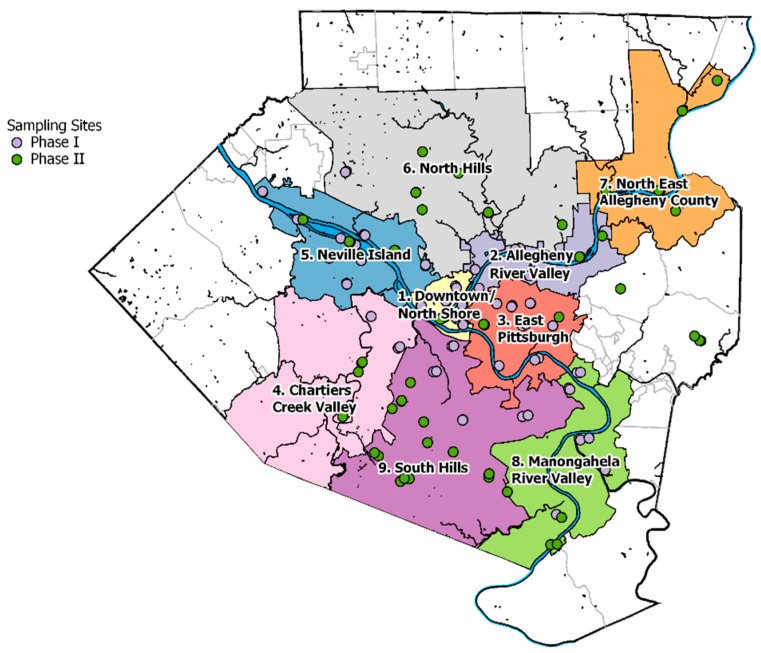
Location of each sampling site and the boundary of each group of sites defined based on proximity and geography. Sites from the two phases of measurement are separated by color. The outer boundary is the political boundary of Allegheny County, PA.

Phase I measurements were conducted from November 2011 to February 2012 for winter and June to August 2012 for summer, respectively. We modified a Ford E-350 cargo van for our field campaign and loaded it with various instruments (Table 1). Forty-two sites were selected in Phase I, and they were mainly located in three domains: (1) the city of Pittsburgh, (2) the industrial zone on Neville Island and the valley around it and (3) the industrial area along the Monongahela River between Braddock and Clairton. Figure 1 shows a map of the sampling locations and Table S1 in the Supplementary Information lists all of the sampling sites. The major point sources in the Neville Island industrial zone included a metallurgical coke plant and chemical industries. The Monongahela River Valley industrial zone is home to the largest metallurgical coke plant in the U.S., an integrated steel mill, and chemical industries. A stratified site selection was used based on three spatial covariates: elevation above sea level, proximity to traffic, and proximity to point sources. Classification for each covariate is binary; sites are either low elevation (in the river valley) or high elevation (on the surrounding hills), low or high traffic, and low or high industrial influence. Details of the site classification are available in Table S1 and Tan et al. [18].

In Phase I, many of the sites that we visited were among streets with moderate to heavy traffic, and on-street parking space was limited during the sampling hours except during the night-time. Thus, during Phase I sampling, the mobile laboratory circled a single block for each hour-long visit to the site, during which four GC samples were collected. This sampling strategy helps avoid self-sampling because the sampling inlet generally experiences a headwind [18]. We did not notice any irregularities in the chromatograms collected during the campaign due to the in-motion sampling method. 

#### 2.1.2. Phase II Measurements

Phase II included both summer and winter sampling. However, due to poor data quality of the GC chromatograms, the summer data did not pass our quality controls and we only report winter data here. Winter sampling was conducted during December 2013 to January 2014. Thirty-six sites were selected in Phase II, among which six were repeats from Phase I. Phase II sites expanded our spatial coverage to suburban Allegheny County around North Hills and South Hills areas (Figure 1), where the local traffic and small industrial facilities tend to be the main sources. We deployed a new Nissan NV 2500 HD in Phase II with the same GC unit as in Phase I. Around most of the sites the traffic volume was significantly lower compared to sites from Phase I, and street parking space was usually available. We thus sampled with the mobile laboratory parked at curbside during Phase II. Self-sampling was avoided by placing an 8 m long tube on the vehicle exhaust and placing it downwind of the mobile laboratory [33,34]. As in Phase I, each site was visited three times per season, once each in the morning, evening, and night-time periods. 

### 2.2. Data Processing

**Figure 2:**
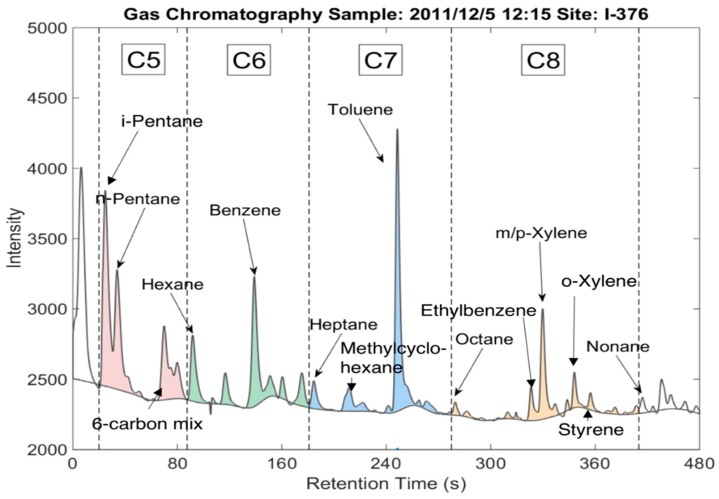
Example chromatogram. Speciated VOC compounds are marked at their peaks. Total VOC concentrations are calculated based on the area between the chromatogram signal and the fitted baseline, starting from i-pentane to everything before nonane.

Each 15 min GC sample generated a chromatogram that featured peaks representing specific compounds (Figure 2). The peaks were identified using Matlab function “mspeaks”, and the baseline was calculated with Matlab function “msbackadj” with “WindowSize” set at 50 s. Speciated peaks were identified and integrated. Raw signal was converted to concentration based on laboratory calibration tests using known standards. Thirteen compounds were speciated from the peaks, as shown in Figure 2. Identified compounds include BTEX and linear alkanes with five to eight carbon atoms. 

Some peaks in the chromatograms could not be assigned to specific compounds. The concentration of unspeciated VOCs was quantified by integrating all signals above the baseline and applying the average sensitivity of the 13 identified species. We took this approach because the FID response (i.e., μg/area) was approximately constant for each of the identified species. Our analysis implicitly assumes that the unidentified peaks are chemically similar (e.g., are aliphatic or aromatic hydrocarbons) to the 13 identified species, as we would not expect oxygenates to have the same sensitivity in the FID. The total VOC signal is shown as the colored areas in Figure 2. It accounts for all identified and unidentified peaks that fall between i-pentane and nonane on the chromatogram. 

Black carbon (BC) concentrations, which were measured at 1 min resolution, were averaged to the same 15 min period to match the GC sampling cycle. As shown in Table 1, we used different BC instruments in Phase I and Phase II. In each case we used the instrument default mass absorption cross-section to convert light attenuation to BC concentration. The two instruments were co-located prior to the Phase II deployment and we observed quantitative agreement.

### 2.3. Site Aggregation

Tan et al. [35] showed that the mobile sampling scheme adopted in this study can successfully rank-order sites according to annual concentration (e.g., site A has higher concentration than site B). However, the estimate of the annual concentration itself is relatively uncertain. This is because the limited number of sampling hours (6 hours per site) may not be sufficient to capture the full range of meteorological and source/emissions conditions. Increasing the sampling hours by aggregating several sites together can effectively add to the accuracy of the estimate of annual average concentration, at the cost of spatial resolution. We applied two aggregating methods to the data collected in this study and investigate how location and source impact would affect VOC concentrations and the source mix determined from mobile sampling. 

#### 2.3.1. Groups of Sites Based on Proximity and Topography

**Figure 3:**
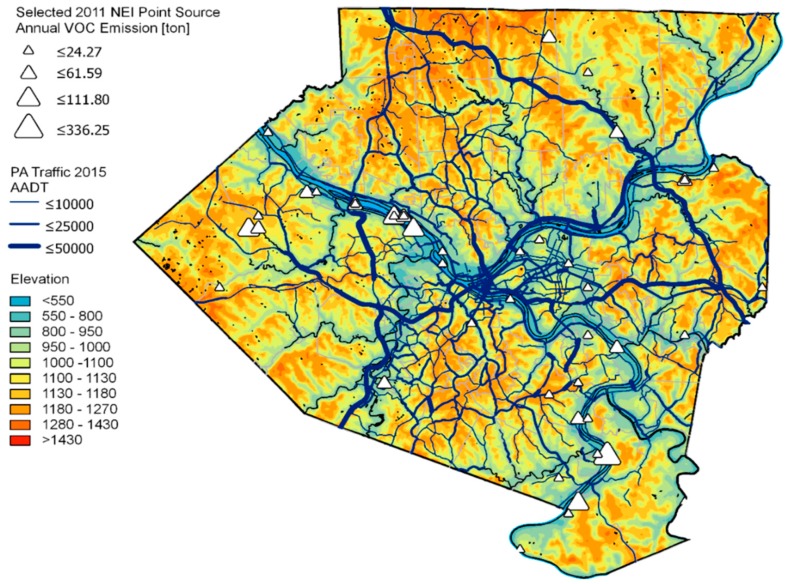
Map of elevation above sea level (in feet), major roads and major VOC point source facilities in Allegheny County. AADT = Annual Average Daily Traffic counts.

We assigned the measurement sites into nine groups based on their proximity to each other and the topographical and land use features in the region (Figure 1). The boundaries of groups shown in Figure 1 follow the municipality boundaries in Allegheny County. Topography features, such as valley and hilltop areas, as well as major traffic and point sources were considered during site grouping (Figure 3). For example, Downtown/North Shore is a valley area surrounded by hills and rivers that features high traffic volumes. East Pittsburgh is a hilly urban area with high traffic but no industrial sources. Neville Island, Monongahela River Valley and Allegheny River Valley are all valley areas that contain major industrial sources; Allegheny River is also a high traffic area. Chartiers Creek Valley has a major highway at the bottom of the valley, which helps accumulate fresh emission in a more restricted space. North Hills and South Hills are both hilly suburban regions with less impact from major roads and major industries. While sites in South Hills are mostly classified as high traffic, sites in North Hills are mostly low traffic suburban background sites with minimal local source impact. Three Phase II sites did not fit into this site grouping scheme and were therefore not assigned to a group.

#### 2.3.2. Types of Sites Based on Land-Use Covariates

Land-use covariates are commonly used as predictors of ambient air pollutant concentrations in models such as land-use regression (LUR) [13,31,32,36]. In this study, we select elevation above sea level and traffic volume as the two covariates to stratify the sampling sites and analyze how these factors may contribute to VOC concentrations and source contributions. For each covariate, we stratify the sites into two tiers: an elevation of 850 ft. separates upper land (U) and valley (V), and an annualized average daily traffic (AADT) of 2500 vehicles per day separates the high traffic locations (T) and low traffic locations (with no T indicated). Altogether four types of location are defined (U, U+T, V, and V+T). Details of each site’s group and type information can be found in Table S1. This site grouping method considers all sites, including the three sites not assigned to proximity-based groups in Section 2.3.1.

While we used proximity to point sources during site selection, we do not include it as a stratification variable during analysis. As previously shown by Tan et al [18,34], pollutant concentrations for BC and NO_2_ were not consistently elevated at sites near industrial sources. This is due, in part, to the fact that some of the near-industry sites are upwind of the major point sources under most typical meteorological conditions. 

### 2.4. Source Identification Techniques 

Concentration ratios are commonly used as signatures to analyze the impact and contribution from specific sources. For example, the concentration ratio of toluene and benzene differs among major sources (gasoline vehicles, diesel vehicles, and industrial emissions) and is widely used to infer the contribution from these sources [37,38,39]. The ratio-ratio plot is a technique that plots the concentration of two species normalized by a third, more stable species, so that essentially three ratios are plotted on a single two-dimensional figure. Ratio-ratio plots allow each source profile to be represented as a single point on a figure [40]. An ambient sample can be explained as a combination of two sources if it falls on the line that connects the two source profiles on the plot, and can be explained by three or more sources if it falls on the plane that is bounded by those sources. On the ratio-ratio plot, the closer an ambient sample is to a source profile, the more that source contributes to the sample [40].

In this study, we use two sets of ratio-ratio plots to help differentiate the impact from specific sources: (1) toluene and benzene concentrations normalized by black carbon (BC) to differentiate gasoline vehicle, diesel vehicle, and industrial sources, and (2) toluene and i+n-pentanes concentrations normalized by benzene to differentiate vehicular tailpipe emission from evaporative fuel and solvent emissions. While the ratio-ratio plot is effective as a visualization tool to provide evidence of source impacts, it is not capable of precise quantification of source impacts. In this study, we use PMF for source apportionment.

PMF (positive matrix factorization) is a statistical approach commonly used for source apportionment of speciated and time-resolved datasets [41,42]. The basic concept of PMF is described in Equation (1). The concentration matrix *x*, which contains *i* observations and *j* species, can be approximated as the linear combination of sources. Each source has a source profile (*f*) describing the mix of pollutant emissions from the source and a source strength (mass contribution) *g*.

(1) $$x_{ij}=\overset{p}{\underset{k=1}{\sum }}g_{ik}f_{kj}+e_{ij}$$

In PMF, Equation (1) is solved without any source profiles being specified. The algorithm, given a designated number of factors, minimizes objective function Q, which is the residual error (*e*) normalized by the uncertainty of each species. The decision of the number of factors (sources) and the evaluation of the factor profiles are the keys to a successful PMF analysis and a reasonable interpretation of the results. A common way of evaluation is to compare the factor profiles to existing source profiles [43,44,45]. PMF does not assume any knowledge of the sources that impact the collected data and relies on math alone to reach an optimal solution. This simplifies the model because no assumptions of existing sources need to be made (as is what happens in the chemical mass balance model), nor will any potential unknown sources be left out from the analysis. But it also complicates the interpretation in cases when a resolved factor does not resemble any known sources. There can also be variation in the factor profile from the known source profile. In this situation, PMF factors may only represent a generalization of a certain type of source profile but neglect intra-type variations. 

We used EPA PMF version 5.0 in this study. The PMF analysis included thirteen VOC species (Figure 2) and BC; each of these inputs was labeled as “strong” (i.e., not down weighted). We resolve five factors that can be reasonably characterized as relevant sources in the Pittsburgh region. The PMF results also match the trends that are observed from ratio-ratio plot technique, which adds to the credibility to the results from both techniques.

## 3. Measurement Results 

### 3.1. Average Concentration by Geographic Groups

**Figure 4:**
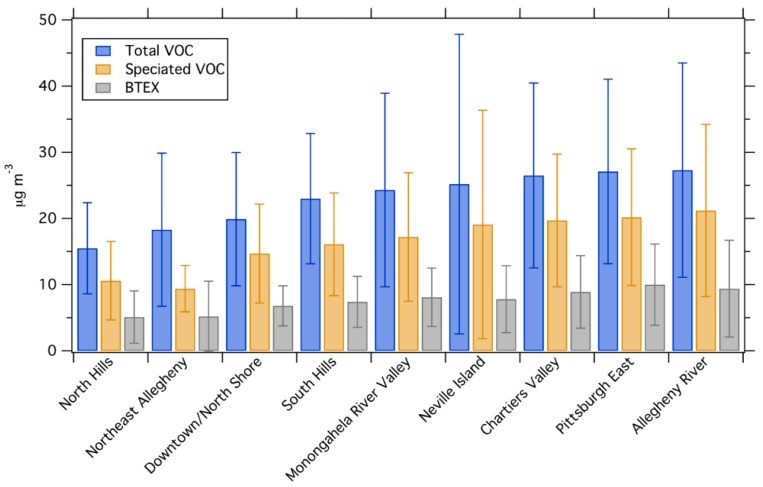
The average concentration of total VOC, speciated VOC (sum of 13 species identified in Figure 2), and BTEX for each group of sites, ranked based on the order of total VOC concentrations. Error bars show one standard deviation.

Figure 4 shows the average concentration of total VOC, speciated VOC and BTEX in each geographic group. Mean concentrations of total VOC, speciated VOC, and BTEX all show approximately a factor of two difference between the groups with the lowest and highest concentrations. 

Concentrations are lowest in North Hills and Northeast Allegheny County. Many of the sites in these areas are low traffic suburban background areas, so the lower VOC concentrations in these areas are consistent with low source activity. The highest concentrations are observed in the Allegheny River Valley, Pittsburgh East, Neville Island, and Chartiers Valley areas, with similar concentrations in all four of these areas. While VOC concentrations are similar in these areas, the land use is not. Pittsburgh East is composed mainly of urban residential neighborhoods with high traffic. Many of the sampling sites in Chartiers Valley and Allegheny River Valley are high traffic sites in the river valleys, though the sites are more suburban than the Pittsburgh East sites. The Neville Island area has several large industrial facilities, including a metallurgical coke oven. 

The Neville Island and Monongahela River Valley areas both have multiple industrial sources and are generally low traffic areas. Due to the industrial emissions, we expected to find the highest VOC and BTEX concentrations in these areas. However, this is not the case. This may suggest that regional traffic is more important for VOCs in Pittsburgh than industrial emissions. Concentrations are most variable in the industrial Monongahela Valley and Neville Island areas; this variability may be the result of transient industrial plumes in those areas.

The Downtown area was surprisingly low in VOCs, especially given that this area has the highest traffic in our sampling domain. This is in contrast to BC and NOx, which Tan et al. [18,34] previously showed have higher concentrations in downtown than other areas. 

Figure 4 shows that the concentration differences across the domain are roughly a factor of two. The VOC spatial variations are smaller than spatial variations of BC in the same domain, comparable to NO_2_, and larger than spatial variations in PM_2.5_ [18,34,35]. However, most differences between site groups are not statistically significant. Table S2 in the Supplementary Information shows results from an ANOVA comparing each site group to the overall dataset. Only the low-source North Hills area has total VOC concentrations that are significantly different than the mean for the domain. Both North Hills and Pittsburgh East are significantly different than the mean for BTEX concentrations.

### 3.2. Average Concentration by Land Use Types

**Figure 5:**
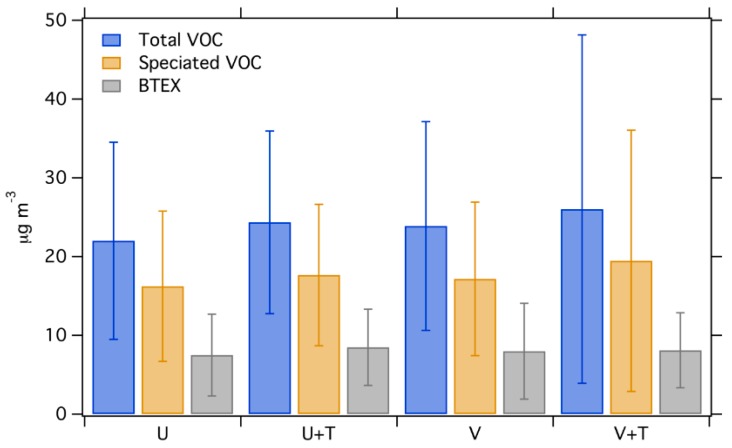
The average concentration of total VOC, speciated VOC and BTEX for each type of site when classified by land use. Error bars show one standard deviation.

Figure 5 shows the average concentration of total VOC, speciated VOC and BTEX among the site types as defined by the stratification variables (e.g., low versus high traffic valley sites). This site aggregation is coarser than the geographic aggregation used above, as more sites fall into each category. The difference among the types are not as pronounced as that among the groups of sites. The strata with the highest concentration (V+T) is only 20% higher than the lowest strata. This method of grouping does show that valley sites have a slightly higher concentration than upper land sites, while high traffic sites have a slightly higher concentration than low traffic sites. However, the differences between site types are small and not statistically significant (Table S2); moving from low- to high-traffic increases BTEX concentrations by less than 1 μg/m^3^. This is in contrast to source-resolved PM components, which we previously showed have statistically significant spatial patterns among land-use classes [22].

### 3.3. Average Concentration by Season

**Figure 6:**
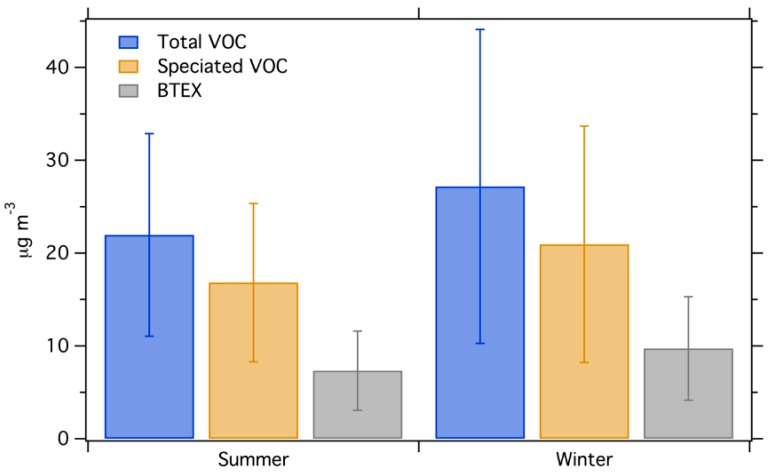
Seasonal variation of total VOC, speciated VOC and BTEX concentrations for Phase I dataset. Error bars show one standard deviation.

Figure 6 shows the concentration of total VOC, speciated VOC and BTEX among all Phase I sites in winter and summer (Phase II is not shown because the summer data failed QA/QC). Concentrations observed in winter are on average 27% higher than summer for all measures. BTEX concentrations are 33% higher in winter. Two possible reasons can be given to explain this difference: (1) lower boundary layer height during night-time in the winter season may lead to accumulation of fresh emissions, (2) vehicle cold-start emissions may be higher in the winter. Cold starts contribute to 80% of total vehicle VOC emissions for modern vehicles, and cold starts may be more intense (e.g., because of colder initial engine and catalyst temperatures) in the winter [46]. Although evaporative emissions are likely more intense in hotter seasons, it does not seem to overwhelm the other factors that favor higher VOC concentrations in winter. Faster photochemical oxidation may also play a role in lower VOC concentrations in the summer months, though this is likely a minor driver for BTEX species that have photochemical lifetimes of hours to days even under summer conditions.

### 3.4. Comparison with Previous Studies

Two VOC monitoring campaigns were conducted in Pittsburgh before this study, and both of them involved stationary-deployed GC systems at selected sites. Millet et al. [47] sampled at a background site located in an urban park near the Carnegie Mellon University (CMU) campus, aiming to capture the urban background concentration level for the city. This sampling site was in the East Pittsburgh area shown in Figure 1. The measurements were conducted in 2001–2002. 

In 2006–2008, Logue et al. [48] measured at the same site as Millet et al as well as at a site in Downtown Pittsburgh and another in Avalon, which is inside the Neville Island industrial area. We select data from one of our downtown sites (6th and Penn, Table S1) to compare with the Downtown site from Logue et al, and one of our Neville Island sites (Bellevue) with Avalon. For this study, we do not have a central site near CMU campus that targets the background concentration, so we select the site with the lowest median concentration for comparison.

Table 2 lists the concentration statistics for benzene, toluene, and m/p-xylene for all the studies and sites described above. In general, concentrations fell from 2001–2002 to present. For benzene, median concentrations at the urban background fell by roughly a factor of three between 2001–2002 and 2006–2008, with an additional 30% reduction between 2006–2008 and this study. Median benzene concentrations Downtown, while higher than the urban background, also fell by roughly 30% between 2006–2008 and this study. These decreases likely reflect reductions in vehicular emissions [49]. Benzene concentrations in the industrially-impacted Neville Island area were higher than Downtown in both 2006–2008 and 2011–2012 and had a larger decrease (~50%) over that time. The additional decrease in benzene concentrations near Neville Island relative to Downtown and the urban background may indicate reductions in industrial emissions in addition to reductions in vehicle emissions. 

The trends in toluene are similar to benzene. Median urban background concentrations fell by more than half between 2001–2002 and 2006–2008, with an additional ~20% reduction between 2006–2008 and this study. Toluene concentrations in both 2006–2008 and 2011–2012 were elevated in Downtown and Neville Island, with higher concentrations in Neville Island. Absolute concentration reductions in these areas from 2006–2008 to 2011–2012 were larger than in the urban background, likely reflecting emission reductions from vehicles and industrial sources.

The temporal trend for median m/p-xylene concentrations is less clear. While there is an overall decrease in the urban background from 2001–2002 to 2011–2012, the 2006–2008 measurements had significantly lower concentrations. Likewise, there is a slight increase in median m/p-xylene in Downtown between 2006–2008 and this study. Given that benzene and toluene concentrations fell with time in all three areas, the drivers for increasing m/p-xylene concentrations are unclear.

## 4. Source Apportionment Analysis

In this section we use ratio-ratio plots and PMF to analyze the source impacts driving the observed VOC spatial patterns. The ratio-ratio plot technique can be considered as a visualization tool that provides a general idea of how the data compare to known source profiles, while PMF is a mathematical tool that attempts reach an optimal solution that provides information of both source profile and contribution.

### 4.1. Ratio-Ratio Plots

**Figure 7:**
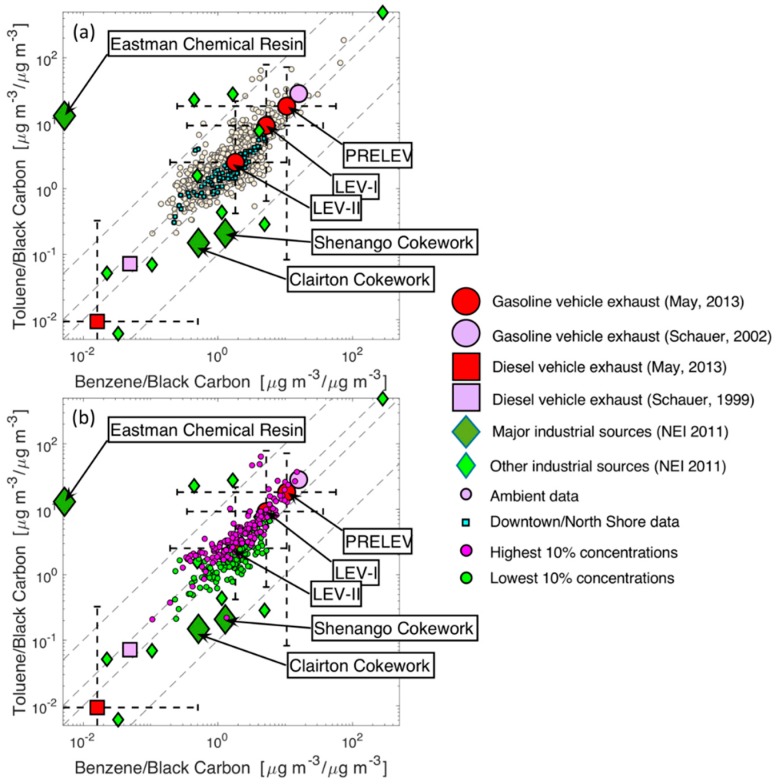
Ratio-ratio plot with benzene and toluene concentrations normalized by Black Carbon (BC). (**a**) All samples (white dots) and samples from Downtown/North Shore (cyan squares) are plotted on a log scale. (**b**) Top 10% highest total VOC concentrations are plotted as magenta circles and lowest 10% total VOC concentrations are plotted as green circles. Gasoline vehicle (large circles) and diesel vehicle (large squares) emission profiles, as well as industrial emission profiles in Pittsburgh (diamonds) are overlaid to visualize the source impacts.

Figure 7 shows ratio-ratio plots of benzene and toluene concentrations, each normalized by black carbon. We expect that gasoline vehicles, diesel vehicles, and industrial emissions are the major sources of these three pollutants in our sampling domain.

Figure 7 shows that most of the data lie along a line bounded by source profiles for gasoline vehicles in the upper right and diesel vehicles in the lower left. This suggests that most of the variation in our observations of benzene and toluene can be described as a mixture of emissions from gasoline and diesel vehicles. However, there is significant scatter in the data along the gasoline-diesel mixing line; for a given benzene/BC ratio, the toluene/BC ratio can vary by up to a factor of four. This scatter can be driven by emissions from industrial sources, which are shown as diamonds in Figure 7. 

Gasoline vehicle emissions have changed with increasingly stringent regulations, and older vehicles, as indicated by the Schauer et al and ‘Pre-LEV’ (pre-1993) source profiles in Figure 7, have higher toluene/BC and benzene/BC ratios than newer LEV-1 (1994–2003) and LEV-II (2004–2013) vehicles. These ratios have changed because of lower VOC emissions from newer vehicles, with roughly constant BC emissions [49]. The gasoline vehicle profiles fall on roughly the same line as our data. Likewise, newer diesel vehicles also have lower toluene/BC and benzene/BC ratios, though this change is driven by reductions in both VOC and BC emissions, especially for newer diesel vehicles equipped with diesel particle filters.

The mean gasoline vehicle age, and thus the emission profile, in Pittsburgh during our sampling falls somewhere between LEV-I and LEV-II [50]. Some of the measurement data are located further towards the upper right corner of Figure 7 than the LEV-I profile. This suggests that there is another emissions source, such as solvent or fuel evaporation, near the upper right corner of the plot; this would be a source with high benzene/BC and toluene/BC ratios. 

The two panels of Figure 7 highlight different subsets of the data as case studies. Figure 7a highlights the data from Downtown/North Shore. Data in this area seem to be more dominated by vehicle emissions than the overall dataset, as the data points align much more closely with the vehicular emission profiles compared to the rest of the data (e.g., there is less spread away from the diagonal line defined by the vehicle profiles for Downtown data compared to the full dataset). Additionally, none of the Downtown data points extend beyond the LEV-I source profile, suggesting that the source with high benzene/BC and toluene/BC ratios is not as important in this area.

Figure 7b shows the samples with the highest and lowest 10% of total VOC concentrations. Examining the distribution of the extreme samples on the ratio-ratio plot can provide an insight into the sources that are responsible for the highest observed concentrations in the region. However, no one source seems to dominate either of these subsets of the data. In general, there is significant overlap in the benzene/BC ratio for these two subsets of the data. The points in the lowest 10% of VOC concentrations tend to have slightly lower toluene/BC, as these points are mainly below the diagonal line defined by the vehicle source profiles. The higher concentration samples tend to stretch toward the top-right corner of the space, suggesting the importance of the source with high benzene/BC and toluene/BC. However, these differences are slight, and there is not an obvious source or set of sources driving high concentrations. Higher concentrations may instead be driven by meteorology (e.g., boundary layer height) with a relatively stable mix of emission sources.

**Figure 8:**
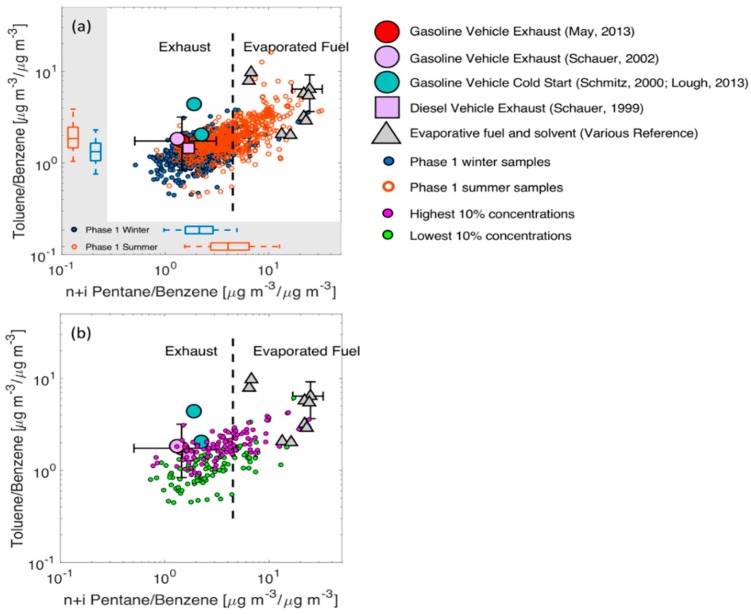
Ratio-ratio plot with toluene and n+i-pentane concentrations normalized by benzene concentrations. (**a**) Phase I winter data are plotted in blue circles and Phase I summer data are plotted in orange circles. The distribution of these ambient data on two axes are also shown in boxplots, with lines representing medians, boxes representing interquartile range, and whiskers representing 5th and 95th percentiles. (**b**) Data with the highest and lowest 10% of total VOC concentrations. Gasoline vehicle (circles) and diesel vehicle (squares) emission profiles, as well as evaporative fuel and solvent emission profiles (grey triangles) are overlaid to visualize the source impacts.

The source with high benzene/BC and toluene/BC that resides in the upper right corner of Figure 7b may be an evaporative emissions source. Figure 8 uses toluene and n+i-pentane, both normalized by benzene, to examine the potential impacts of evaporative emissions. Evaporated fuel has significantly higher (~factor of 10) n+i-pentane/benzene ratios than exhaust and modestly higher toluene/benzene rations (~factor of 2). While there is variability in the evaporated fuel profiles, likely due to the variable fuel composition used in different source profiles [51,52,53], there is a clear distinction between the evaporated fuel and tailpipe emissions profiles.

The data collected from Phase I are plotted in Figure 8a along with the vehicle and evaporative fuel solvent emission profiles, and the different colors show the data from summer and winter separately. We focus on Phase I data in Figure 8a in order to highlight seasonal differences among a common set of sampling sites. The data generally fall between the tailpipe emission and evaporative emission source profiles, suggesting that these two sources can describe most of the observed variability. There is a portion of the data, predominantly in winter, that has lower toluene/benzene and n+i-pentane/benzene than the tailpipe emissions profiles. This may indicate an additional source, or seasonal variations in tailpipe emissions that are not captured in source tests. Comparing the summer and winter data points, summer samples in general have higher ratios of toluene to benzene and pentanes to benzene. Since the VOC concentrations are different in the two seasons, the trend in Figure 8a indicates that the contribution from evaporative sources relative to vehicular emission is more important in summer than in winter. This is a reasonable conclusion since evaporation of VOCs should scale with temperature and therefore be a more important source in the summer.

The data in Figure 8b show the top and bottom 10% of samples in terms of total VOC. As with the analysis of this subset of the data in 7b above, there is not a clear distinction that indicates a particular source driving the higher concentrations. Toluene/benzene ratios are slightly higher in the top 10% of VOC measurements, but there is also significant overlap with the bottom 10% of data. The overall implication of Figure 8 is that evaporative emissions seem to be an important VOC source in this domain, but they do not drive high concentration events.

### 4.2. Source Apportionment with PMF

**Figure 9:**
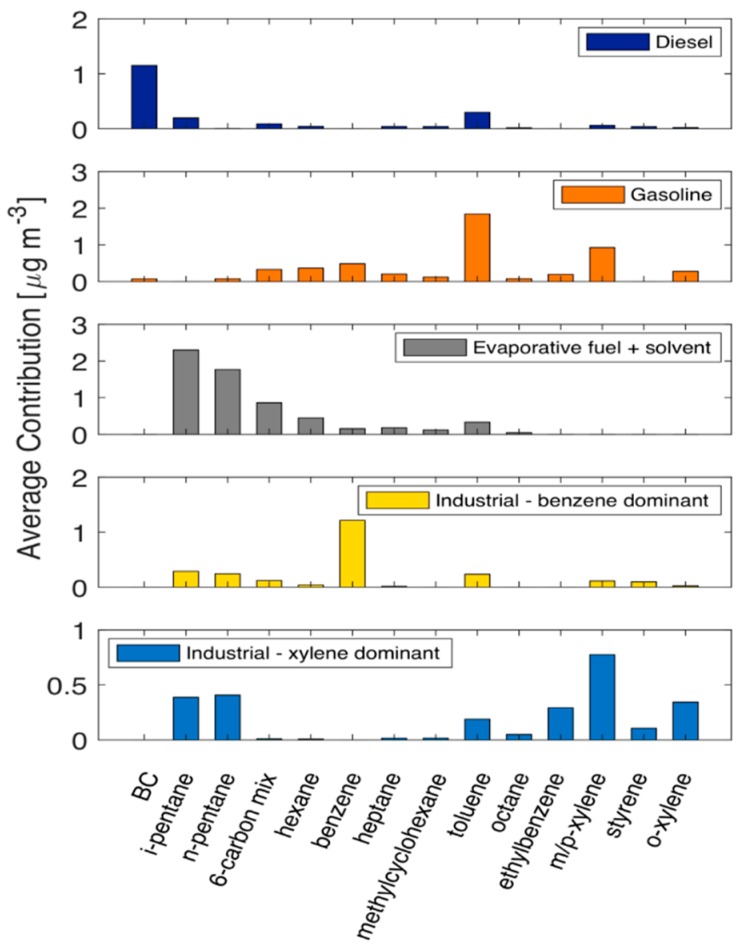
Factor profiles for the five-factor solution of the Positive Matrix Factorization (PMF) model based on the speciated VOC concentrations along with BC concentrations.

Ratio-ratio plots provide a qualitative way to examine the data, but do not provide a quantitative result for source contribution. To quantify the observations from analysis of ratio-ratio plots, we performed positive matrix factorization (PMF) analysis on the speciated VOC dataset from the mobile sampling campaign. We performed PMF analysis on the entire dataset. As shown in Figure 9, we used a five-factor solution to describe our data. The five factors are: diesel vehicles, gasoline vehicles, evaporative fuel + solvent, industrial-benzene dominant, and industrial-xylenes dominant.

Details of the PMF error and sensitivity analyses are included in the Supplementary Information. Figure S1 shows the results of bootstrap analysis. Figure S2 shows tests of rotational ambiguity using Fpeak. Lastly, there were no instances of source swaps during displacement analysis. These tools therefore show that our PMF solution is stable.

The diesel vehicle factor contributes to the majority of the measured BC concentration (94%) but less than 10% for most of the VOC compounds. Generally speaking, diesel is not a major contributor to VOC concentrations [49,54]. For the whole dataset, the diesel vehicle factor contributes 5% of speciated VOC. Compared to the diesel vehicle profiles from source tests, our ambient diesel factor is richer in toluene (normalized by BC) by a factor of four (0.29 vs 0.07 from Schauer et al [54]). There is also zero benzene attributed to the ambient diesel factor, which makes it impossible to plot this factor in the ratio-ratio plots in the previous section. However, the diesel factors obtained from source tests lie well outside the range of our dataset (e.g., we have no data points that look like “pure diesel” in Figure 7). Thus, we should not expect our PMF-derived diesel source profile to exactly match source tests. Any source profile with both toluene/BC and benzene/BC less than ~0.3 should be sufficient to define the lower-left limit of the mixing space shown in Figure 7.

The gasoline vehicle factor contributes on average 64% of toluene concentration and more than 40% of all xylenes, ethylbenzene and linear alkanes, except pentanes. It contributes 28% of the total speciated VOCs. The contribution to pentanes from this factor is almost negligible, which is inconsistent with published gasoline vehicle emission profiles [7,49]. The toluene-to-benzene ratio of the ambient gasoline vehicle factor is around 4, which is higher than typical emission profiles (usually around 2). This suggests that the gasoline factor may overestimate the contribution to toluene and obviously underestimate the contribution to pentanes. However, this is the factor profile that most closely resembles the published gasoline profiles, so we define it as a gasoline vehicle factor here. 

The evaporative fuel + solvent factor in this solution resembles headspace gasoline vapor profiles [55] but may also include other sources of evaporative VOCs. It contributes around 70% of the pentanes and around 50% of all other straight chain alkanes except octane. This factor contributes 36% of the total speciated VOCs. For this factor, the pentanes-to-toluene ratio is 12 and the pentanes-to-benzene ratio is 26. These ratios are consistent with those found in evaporated fuel [51,52,53]. However, since our gasoline factor profile is depleted in pentanes, it is possible this factor over-attributes pentanes, and thus total VOCs, to fuel evaporation. The ambient dataset contains a combination of complicated vehicle operations, which may blur the boundary of the gasoline vehicle profile and the evaporative profile, making PMF inefficient at separating them. 

The industrial-benzene dominant factor is half benzene and half other compounds. It contributes to 65% of average benzene concentration from the mobile dataset. Notably, it also contributes to 40% of styrene, which is a known emission from chemical plants located in Allegheny County [48,56]. For the whole dataset, the industrial-benzene factor contributes to 13% of total speciated VOC. This factor is likely a Pittsburgh specific factor, as the benzene emission from coke plants in the area are well documented [56]. The benzene factor is on average the second smallest contributor to total VOC concentration (only greater than diesel vehicles). Nevertheless, this factor is still important from a public health perspective as it is the dominant contributor to benzene concentrations.

The industrial-xylene dominant factor features high xylenes concentrations along with considerable pentanes contribution. This factor does not get resolved if the PMF model only includes data from Phase I samples, indicating that the major sources for this xylene emission may be scattered within the Phase II sampling domain (mainly in the suburban areas). It also contributes to 45% of styrene, which suggests industrial process. For the whole dataset, the xylene factor contributes to 15% of total speciated VOC.

We also investigated PMF solutions with four and six factors. The four-factor solution has a higher residual than the five-factor solution. The four-factor solution maintains the diesel, gasoline vehicle, evaporative, and benzene factors, and redistributes xylene across those four factors. Overall, the diesel, gasoline, and evaporative factors show better agreement with published source profiles for our five-factor solution than the four-factor solution. The six-factor solution resembles the five-factor solution but seems to over interpret the data by creating a toluene-dominant factor from parts of the gasoline vehicle factor. The result is that both the new toluene factor and the new gasoline vehicle factors in the six-factor solution do not resemble any typical sources we are aware of either in Pittsburgh or nationally. Thus, we consider the five-factor solution as the best solution.

### 4.3. Variation of Factor Contributions among Groups and Types of Sites

**Figure 10:**
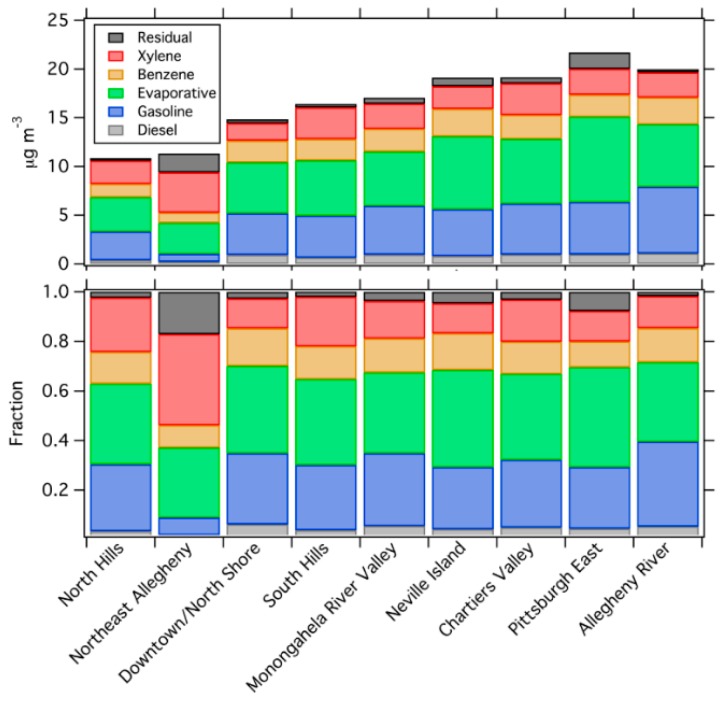
The concentrations and fractions of each PMF factor in each geographic group of sites.

**Figure 11:**
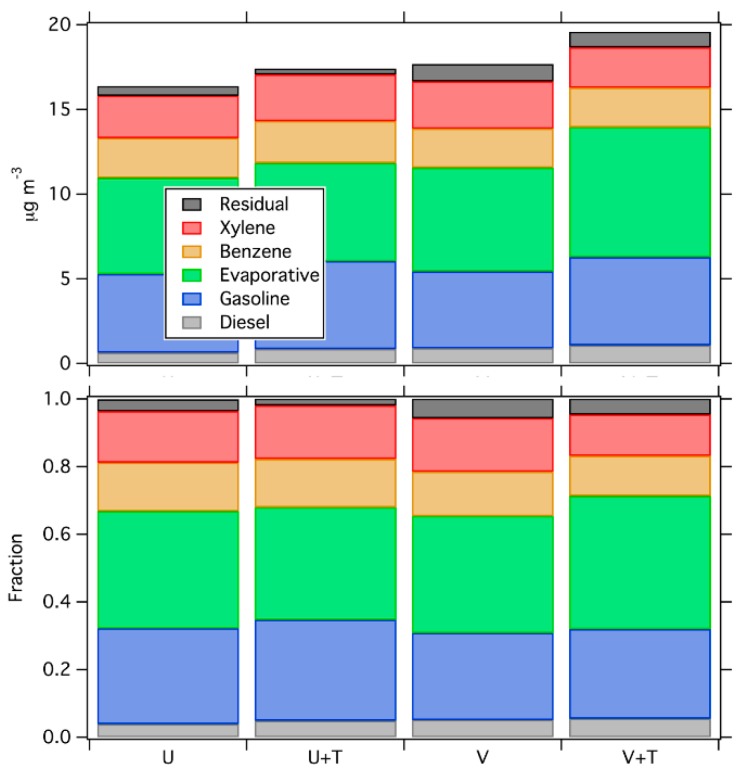
The concentrations and fractions of each factor in each type of site. The source mix is similar across the various land uses.

Figure 10 and Figure 11 show the PMF source apportionment, both in terms of absolute mass concentration and relative contribution, across the geographic groups and site strata. In general, there is not a significant difference in the breakdown among factors for most groups and site types. While the total concentrations change, the fractional contribution of each source is relatively consistent in each area. There are a few small spatial variations, such as less diesel influence in the low-traffic North Hills area, but in general the source mix is similar across the different areas.

The only geographic group that is significantly different from the others is Northeast Allegheny, which features much higher contribution from the xylene and evaporative factors, and much less from the gasoline vehicle factor. This area also has a high residual, suggesting that our PMF solution does a poorer job of resolving sources in this area than other areas. There are several possible reasons why the Northeast Allegheny area appears different than other areas: (1) This area has a smaller sample size, which may impact the apportionment. (2) There may be significant emission of xylenes and evaporative fuel and solvents in this region that do not get mixed into other regions, possibly due to the prevailing southwesterly wind direction. (3) There may be an additional source, which drives the high residual but is poorly resolved in both the five- and six-factor PMF solutions. 

Comparing the sites by land use (Figure 11) also shows relatively consistent source patterns. High traffic sites do not have significantly greater contributions from the diesel and gasoline sources than low traffic sites. Thus, the PMF result suggests that the VOC source mix in Pittsburgh is relatively spatially homogeneous, even though land use changes drastically across our sampling domain. 

One of the limitations of this analysis is that the temporal and spatial resolutions, while high compared to typical air quality studies, may not be sufficient to capture all of the spatial variations. While plumes emitted from industrial sources can spread over kilometer-scale distances with timescales of several hours, many other sources emit more transient plumes. Plumes from individual high emitting vehicles or restaurants cannot be detected at a 15 min resolution, and instead require sampling frequencies of ~1 min or faster [18,22]. Higher time resolution in the speciated dataset helps the PMF model to differentiate the transient plume events from the background concentration in busy areas. The combination of averaging the GC sample over 15 min along with our spatial aggregation may artificially smooth out spatial variations in source impacts. 

Additionally, we performed PMF analysis on the entire dataset. This means that the five factors shown in Figure 9 represent average factors that describe our data in both time and space. An advantage of this approach is that we identify a common set of sources for the entire domain (e.g., we do not derive different gasoline vehicle profiles for each geographic area), which improves the overall robustness of the PMF solution [57]. However, the combination of low time resolution data and performing PMF on the entire dataset may artificially dampen spatial variations in source-resolved VOCs. Higher time resolution data, or additional data points that allow us to perform separate PMF analyses for each geographic area, may reveal more spatial variation. We previously showed that 1 min aerosol mass spectral data revealed large gradients in traffic-related PM emissions [22,58,59,60]; similar spatial structure may be revealed with high time resolution VOC data.

## 5. Conclusions

We utilized a mobile sampling platform with an on-board GC system to collect ambient VOC concentration data in an urban environment. The mobility of this platform and the relatively short GC cycle enabled us to obtain a spatially and temporally resolved dataset. We observed spatial variations in total and speciated VOCs of roughly a factor of two across our sampling domain. We performed two sets of source-oriented analyses to determine the major VOC sources. The dominant VOC contributors are gasoline vehicle emission and evaporative emissions of fuel and solvents. Seasonal analysis shows that evaporative emissions are a larger contributor in summer than winter. Industrial emissions are also important for benzene and m/p-xylene. While we expected the impacts of the industrial sources to be spatially variable, they are instead spatially homogeneous and act more like regional background sources. Diesel vehicles are found to be a minor contributor to VOC concentrations. These findings are important for understanding of the source impact to VOC concentrations in urban environments and can be helpful for public policy decision making and future research planning.

The main limitation of this study is the sample size for each site we visited. The small number of sampling visits to each site prevents us from being able to use PMF on each group of sites. In order to achieve reliable estimates, we had to sacrifice some spatial resolution in the analysis. This may partly lead to the apparent conclusion that the source mix for the whole sampling domain is largely homogeneous. The 15 min averaging time may also have eliminated the temporal variability of the speciated data, which is a desired data feature for source apportionment analysis. To improve on these limitations, future studies should focus on (1) high time resolution measurements and (2) sufficient sampling so that source apportionment can be conducted separately for each spatial sub-domain as needed. 

## Figures and Tables

**Table 1:** General information for the two phases of measurements in this study.

	Phase I	Phase II
Number of sites	42	36 (6 repeat sites from Phase I)
Sampling period		
Summer	November 2011 to February 2012	June 2013 to August 2013
Winter	June 2012 to August 2012	December 2013 to January 2014
VOC ^a^ instrument	Chromatotec GC-FID BTX-866	
BC ^b^ instrument	Multi-Angle Absorption Photometer (MAAP)	Magee Scientific Aethalometer (AE-33)

^a^ Volatile Organic Compound. ^b^ Black Carbon.

**Table 2:** Comparison of measurement results with previous studies. Due to the fact that there is no urban background site measurement near CMU campus, we select the site with lowest median concentration (site name noted in parentheses) for comparison to previous studies.

	Downtown			Neville Island			Urban Background		
	**Benzene (μg/m^3^)**								
	**Mean**	**Median**	**Interquartile**	**Mean**	**Median**	**Interquartile**	**Mean**	**Median**	**Interquartile**
Millet 2005	N/A			N/A			N/A	1.04	0.85 to 1.30
Logue 2009	1.23	0.95	0.61 to 1.53	2.67	1.68	0.92 to 3.46	0.52	0.36	0.23 to 0.56
This Study	0.75	0.68	0.62 to 0.90	1.96	0.76	0.57 to 3.67	0.29	0.29	0.20 to 0.37
							(Fox Chapel)		
	**Toluene (μg/m^3^)**								
	**Mean**	**Median**	**Interquartile**	**Mean**	**Median**	**Interquartile**	**Mean**	**Median**	**Interquartile**
Millet 2005	N/A			N/A			N/A	1.40	1.06 to 2.08
Logue 2009	2.20	1.49	0.82 to 2.69	4.01	2.45	1.55 to 5.14	0.89	0.62	0.34 to 1.16
This Study	1.02	0.92	0.88 to 1.20	1.03	1.00	0.62 to 1.45	0.55	0.55	0.35 to 0.74
							(Creighton-Tarentum)		
	**m/p-Xylene (μg/m^3^)**								
	**Mean**	**Median**	**Interquartile**	**Mean**	**Median**	**Interquartile**	**Mean**	**Median**	**Interquartile**
Millet 2005	N/A			N/A			N/A	0.84	0.56 to 1.26
Logue 2009	1.59	0.93	0.47 to 1.88	2.56	1.48	0.83 to 3.40	0.20	0.14	0.07 to 0.28
This Study	1.27	1.19	0.76 to 1.81	0.95	0.60	0.43 to 1.57	0.95	0.60	0.43 to 1.57
							(Bellevue)		

## Supplementary Materials

The following are available online at https://www.mdpi.com/1660-4601/16/9/1632/s1, Figure S1: Results of PMF bootstrap analysis, Figure S2: Results of PMF Fpeak analysis, Table S1: Site information. This table lists the site name, the phase of measurement campaign, the type of site based on GIS covariates, the traffic volume (AADT), and the group of sites based on proximity and geography, Table S2: ANOVA results.
