# Supplementary material for: Quantifying Urban Spatial Variations of Anthropogenic VOC Concentrations and Source Contributions with a Mobile Sampling Platform
Source: Int J Environ Res Public Health. 2019 May 10;16(9):1632. doi: 10.3390/ijerph16091632 (PMC6539943; doi:10.3390/ijerph16091632)
Supplement: Supplementary file 1 [file ijerph-16-01632-s001.pdf]

Quantifying urban spatial variations of anthropogenic VOC concentrations and source contributions with a mobile sampling platform

Peishi Gu, Timothy R. Dallmann, Hugh Z. Li, Yi Tan, Albert A. Presto

Supplemental Information

Table S1. Site information. This table lists the site name, the phase of measurement campaign, the type of site based on GIS covariates, the traffic volume (AADT), the group of sites based on proximity and geography. Land use classes are defined as follows: U = upland (high elevation), V = valley, T = high traffic, P = close to point sources.

Site	Phase	Type	AADT	Group	Lat.	Long.	Elev. (ft.)
Hill District	1	U	2745	Pittsburgh East	40.444	-79.978	999
Ohio	1	U	2560	North Hills	40.553	-80.100	1202
Ben Avon	1	U+P	1969	Neville Island	40.505	-80.083	823
South Brook	1	U	1491	South Hills	40.402	-80.013	1153
Highland Park	1	U	1184	Allegheny River	40.474	-79.917	1043
West Mifflin	1	U+P	715	Monongahela	40.390	-79.877	1157
Coraopolis	1	U+P	240	Neville Island	40.488	-80.116	1116
Lincoln Place	1	U	100	South Hills	40.368	-79.923	1145
McKees Rocks	1	U+P	100	Neville Island	40.486	-80.085	1103
Observatory	1	U	100	Neville Island	40.482	-80.021	1219
Spring Hill	1	U	100	Downtown/North Shore	40.466	-79.990	1013
Wilkinsburg	1	U	100	Pittsburgh East	40.437	-79.893	907
Walnut St	1	U+T	14175	Pittsburgh East	40.452	-79.933	920
ACHD Forbes	1	U+T+P	12756	Pittsburgh East	40.437	-79.961	919
Fifth Ave	1	U+T+P	12342	Pittsburgh East	40.454	-79.915	954
Brentwood	1	U+T	11653	South Hills	40.366	-79.983	1224
CVS	1	U+T	10882	Pittsburgh East	40.453	-79.949	904
Flag Plaza	1	U+T	9586	Downtown/North Shore	40.442	-79.989	859
Squirrel Hill	1	U+T+P	6359	Pittsburgh East	40.436	-79.923	1122
Allentown	1	U+T	5761	South Hills	40.421	-79.994	1136
I-376	1	U+T	5333	Chartiers Valley	40.420	-80.048	1169
Clairton	1	U+T+P	2940	Monongahela	40.294	-79.889	935
Patricia Park	1	U+T	2753	Neville Island	40.469	-80.100	1163
North Shore	1	V	2275	Downtown/North Shore	40.452	-80.025	757
Glassport	1	V	2175	Monongahela	40.323	-79.892	762
Millvale	1	V	2135	Allegheny River	40.479	-79.971	732
Neville background	1	V+P	1719	Neville Island	40.516	-80.145	720
Braddock Library	1	V	1315	Monongahela	40.401	-79.868	783
Greenwood	1	V	637	Chartiers Valley	40.444	-80.075	798
Blair	1	V	100	Pittsburgh East	40.406	-79.947	759
Waterfront	1	V	100	Pittsburgh East	40.412	-79.907	749
Downtown	1	V+T	11198	Downtown/North Shore	40.443	-80.002	735
Neville Chemicals	1	V+T+P	7352	Neville Island	40.503	-80.106	721
Aspinwall	1	V+T	7301	Allegheny River	40.490	-79.903	745
21st Street	1	V+T+P	5549	Downtown/North Shore	40.453	-79.983	738
a15219	1	V+T	5365	Downtown/North Shore	40.438	-79.983	820
PGH North	1	V+T	4964	Allegheny River	40.495	-79.926	739
a15212	1	V+T	4510	Downtown/North Shore	40.455	-80.000	780
Sewickley	1	V+T	4430	Neville Island	40.537	-80.184	759
McKeesport #1	1	V+T	4367	Monongahela	40.351	-79.865	769
McKeesport #2	1	V+T	4243	Monongahela	40.328	-79.840	746
36th Street	1	V+T	4066	Allegheny River	40.465	-79.967	740

Fox Chapel	2	U	100	North Hills	40.513	-79.883	921
Baldwin	2	U	100	South Hills	40.349	-80.018	1197
Bethel Park 2	2	U	100	South Hills	40.322	-80.037	1196
Monroeville	2	U	100		40.425	-79.744	1154
Plum	2	U+P	100	Northeast Allegheny	40.523	-79.769	1045
Ross Twp	2	U	100	North Hills	40.537	-80.030	1201
Shaler 2	2	U	100	North Hills	40.552	-79.987	1208
Shaler Twp	2	U+P	100	North Hills	40.522	-79.958	1013
Upper St. Clair	2	U	100	South Hills	40.341	-80.072	1140
ACHD	2	U+T	12700	Pittsburgh East	40.438	-79.962	924
Mt. Lebanon-St. Clair	2	U+T	8080	South Hills	40.374	-80.054	1093
McCandless Twp	2	U+T	7476	North Hills	40.568	-80.024	1139
Bethel Park	2	U+T	6129	South Hills	40.331	-80.022	1103
Natrona	2	U+T+P	5941	Northeast Allegheny	40.621	-79.727	999
Rodi Road	2	U+T	5733		40.465	-79.824	1208
Bridgeville	2	U+T+P	5503	Downtown/North Shore	40.371	-80.101	883
Castle Shannon	2	U+T	5215	South Hills	40.364	-80.022	1075
Pleasant Hills	2	U+T	5057	South Hills	40.325	-79.957	1216
Dormont	2	U+T	4869	South Hills	40.394	-80.036	1222
Bellevue	2	U+T	4680	Neville Island	40.494	-80.052	966
Mt. Lebanon	2	U+T	3542	South Hills	40.380	-80.045	1196
Jefferson Hills	2	U+T	3531	South Hills	40.312	-79.938	934
Neville-Shenango	2	V+P	3380	Neville Island	40.500	-80.096	727
Neville-West	2	V+P	2507	Neville Island	40.516	-80.144	738
Creighton-Tarentum	2	V	2381	Northeast Allegheny	40.598	-79.763	757
West Elizabeth	2	V+P	1047	Monongahela	40.272	-79.895	738
Verona	2	V	918	Allegheny River	40.504	-79.843	747
Elizabeth	2	V+P	358	Monongahela	40.272	-79.888	738
Springdale	2	V+P	120	Northeast Allegheny	40.538	-79.785	770
Blawnox	2	V+P	100	Allegheny River	40.488	-79.865	746
Heidelberg	2	V	100	Chartiers Valley	40.402	-80.088	802
Carnegie	2	V+T	10993	Chartiers Valley	40.409	-80.084	771
6th & Penn	2	V+T+P	9068	Downtown/North Shore	40.444	-80.001	735
Harmar	2	V+T	9019	Northeast Allegheny	40.539	-79.839	753

Table S2. ANOVA results.

ANOVA comparing each group with all samples (total average)			
**p-value**	**Total VOC**	**ID VOC**	**BTEX**
Allegheny River	0.215	0.077	0.158
Chartiers Valley	0.465	0.413	0.484
Downtown/North Shore	0.061	0.090	0.089
Monongahela	0.889	0.858	0.961
Neville Island	0.584	0.371	0.800
North Hills	0.014	0.007	0.014
Northeast Allegheny	0.241	0.045	0.094
Pittsburgh East	0.126	0.083	0.008
South Hills	0.645	0.375	0.433

ANOVA comparing each type with all samples (total average)			
**p-value**	**Total VOC**	**ID VOC**	**BTEX**
U	0.233	0.286	0.362
U+T	0.805	0.935	0.413
V	0.970	0.888	0.909
V+T	0.355	0.266	0.993

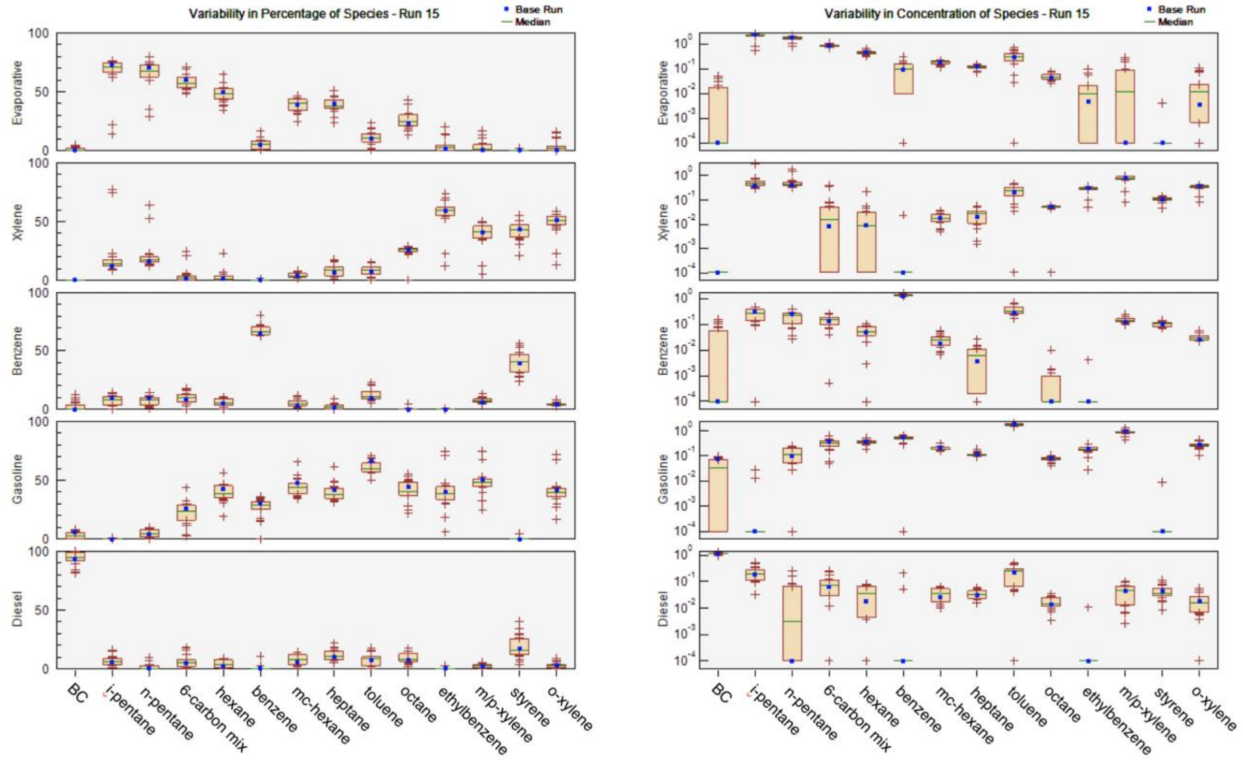

Figure S1. Results of PMF bootstrap analysis

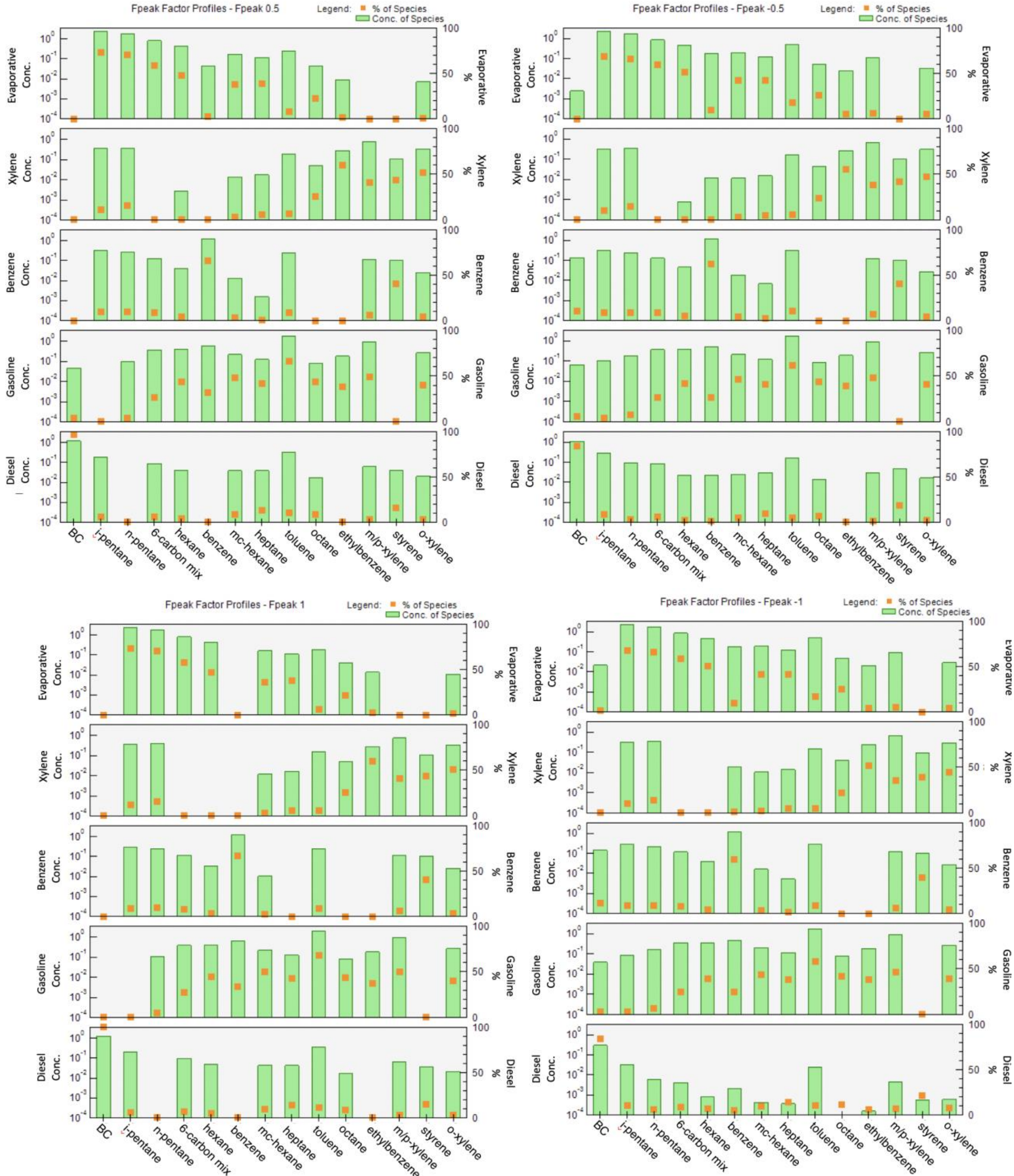

Figure S2. Results of PMF Fpeak analysis.
